# Diaqua­bis­(4-carb­oxy-2-ethyl-1*H*-imidazole-5-carboxyl­ato-κ^2^ *N* ^3^,*O* ^4^)cadmium dihydrate

**DOI:** 10.1107/S1600536811021428

**Published:** 2011-06-11

**Authors:** Gang Zhang, Yong Wang

**Affiliations:** a Department of Chemistry and Chemical Engineering, Henan University of Urban Construction, Pingdingshan, Henan, People’s Republic of China; b Department of Chemical Engineering, Henan Polytechnic Institute, Nanyang 473009, People’s Republic of China

## Abstract

The asymmetric unit of the title compound, [Cd(C_7_H_7_N_2_O_4_)_2_(H_2_O)_2_]·2H_2_O, consists of one Cd^II^ ion, one 4-carb­oxy-2-ethyl-1*H*-imidazole-5-carboxyl­ate anion, one coordinated water mol­ecule and one lattice water mol­ecule. The Cd^II^ ion lies on a twofold axis, and is hexa­coordinated by four O atoms from water mol­ecules and carboxyl­ate groups and two N atoms from two imidazole rings, in a distorted octa­hedral arrangement. An extensive framework of N—H⋯O and O—H⋯O hydrogen bonds with the participation of coordinated and free water mol­ecules is found in the crystal structure, which contributes to the formation of a three-dimensional structure.

## Related literature

For coordination polymers built up from related imidazole–carboxyl­ate ligands, see: Li *et al.* (2011 ▶); Wang *et al.* (2008 ▶); Zhang *et al.* (2010 ▶); Tian *et al.* (2010 ▶). For a related Cd^II^ complex based on the ligand 5-carb­oxy-2-methyl-1*H*-imidazole-4-carboxyl­ate, see: Nie *et al.* (2007 ▶).
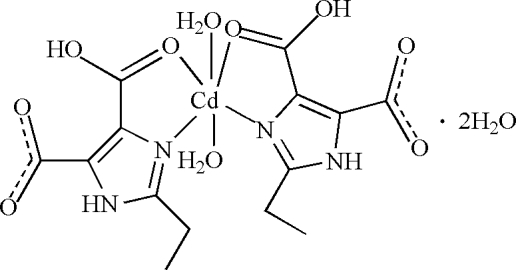


## Experimental

### 

#### Crystal data

[Cd(C_7_H_7_N_2_O_4_)_2_(H_2_O)_2_]·2H_2_O
*M* *_r_* = 550.76
Monoclinic, 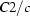

*a* = 9.844 (2) Å
*b* = 17.084 (3) Å
*c* = 12.855 (3) Å
β = 102.21 (3)°
*V* = 2113.0 (8) Å^3^
*Z* = 4
Mo *K*α radiation
μ = 1.10 mm^−1^
*T* = 293 K
0.30 × 0.25 × 0.18 mm

#### Data collection

Bruker SMART 1000 CCD area-detector diffractometer
Absorption correction: multi-scan (*SADABS*; Bruker, 2004 ▶) *T*_min_ = 0.733, *T*_max_ = 0.826
8379 measured reflections
1898 independent reflections
1560 reflections with *I* > 2σ(*I*)
*R*_int_ = 0.042

#### Refinement

*R*[*F* ^2^ > 2σ(*F* ^2^)] = 0.028
*wR*(*F* ^2^) = 0.072
*S* = 1.23
1898 reflections
142 parameters
6 restraints
H-atom parameters constrained
Δρ_max_ = 0.77 e Å^−3^
Δρ_min_ = −0.78 e Å^−3^

### 

Data collection: *SMART* (Bruker, 2004 ▶); cell refinement: *SAINT* (Bruker, 2004 ▶); data reduction: *SAINT*; program(s) used to solve structure: *SHELXS97* (Sheldrick, 2008 ▶); program(s) used to refine structure: *SHELXL97* (Sheldrick, 2008 ▶); molecular graphics: *SHELXTL* (Sheldrick, 2008 ▶); software used to prepare material for publication: *SHELXTL*.

## Supplementary Material

Crystal structure: contains datablock(s) I, global. DOI: 10.1107/S1600536811021428/bh2359sup1.cif

Structure factors: contains datablock(s) I. DOI: 10.1107/S1600536811021428/bh2359Isup2.hkl

Additional supplementary materials:  crystallographic information; 3D view; checkCIF report

## Figures and Tables

**Table 1:** Hydrogen-bond geometry (Å, °)

*D*—H⋯*A*	*D*—H	H⋯*A*	*D*⋯*A*	*D*—H⋯*A*
O3—H3⋯O2	0.81	1.66	2.468 (4)	172
O2*W*—H4*W*⋯O4	0.84	2.16	2.904 (4)	147
O2*W*—H3*W*⋯O1^i^	0.84	2.08	2.874 (4)	157
O1*W*—H1*W*⋯O2^i^	0.84	1.97	2.788 (4)	165
O1*W*—H2*W*⋯O1^ii^	0.84	2.01	2.768 (3)	150
N1—H9⋯O2*W*^iii^	0.91	1.86	2.771 (4)	177

Symmetry codes: (i) 
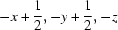
; (ii) 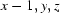; (iii) 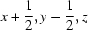.

**Fig. 1.**
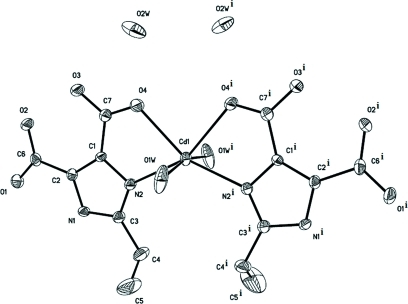
The structure of the title compound, showing the atomic numbering scheme with 30% probability displacement ellipsoids [Symmetry codes i: -x, y, 1/2-z].

## supplementary crystallographic information

### Comment

Self-assembly of supramolecular architectures based on imidazole carboxylate ligands has draw much attention during recent decades. To the best of our knowledge, coordination polymers based on 2-ethyl-4,5-imidazolecarboxylate have been rarely reported so far (Wang *et al.*, 2008; Zhang *et al.*, 2010; Li *et al.*, 2011; Tian *et al.*, 2010). Herein we report the synthesis of the title compound by the reaction of cadmium nitrate with 2-ethyl-4,5-imidazoledicarboxylic acid (H_3_EIDC) in an aqueous solution under hydrothermal conditions, and its crystal structure.

The title compound, [Cd(C_7_H_7_N_2_O_4_)_2_(H_2_O)_2_].2H_2_O, differs from the Cd(II) complex based on the similar ligand 5-carboxy-2- methyl-1*H*-imidazole-4-carboxylate, where the Cd(II) ion is six-coordinated in a centrosymmetric arrangement (Nie *et al.*, 2007). As depicted in Fig. 1, the title complex has two symmetrical coordination water molecules, two interstitial water molecules and two 4-carboxy-2-ethyl-1*H*-imidazole-5-carboxylate ligands (H_2_EIDC). The Cd(II), placed on a 2-fold axis, is surrounded by two terminal water molecules, two N atoms and two O atoms from two different H_2_EIDC ligands, forming a distorted octahedral coordination environment.

One solvent water molecule completes the asymmetric unit, and forms hydrogen bonds with the imidazole N atom (N1), the carboxylic O atom (O4) and the O atom from the coordinated water molecule (O1W), whose distances and angles are shown in Table 1. Each H_2_EIDC ligand is bonded to Cd(II) ion in a chelating mode. A three-dimensional supramolecular structure is consolidated by intermolecular hydrogen-bonding (N—H···O and O—H···O) and intramolecular hydrogen-bonding O—H···O.

### Experimental

A mixture of Cd(NO_3_)_2_ (0.5 mmol, 0.120 g) and 2-ethyl-1*H*-imidazole-4,5-dicarboxylic acid (0.5 mmol, 0.95 g) in 15 ml of H_2_O solution was placed in a 23 ml Teflon-lined reactor, which was heated to 423 K for 2 days, and then cooled to room temperature at a rate of 10 K h^-1^. Crystals of the title compound were obtained by slow evaporation of the solvent at room temperature.

### Refinement

The carboxyl H atom H3 was located in a difference map but refined as riding on the parent O atom with O3—H3 = 0.81 Å and *U*_iso_(H3) = 1.5 *U*_eq_(O3). Carbon and nitrogen bound H atoms were placed at calculated positions and were treated as riding on the parent C or N atoms with C—H = 0.96 (methyl), 0.97 (methylene) and N—H = 0.91 Å, *U*_iso_(H) = 1.2 or 1.5 *U*_eq_(C, N). H atoms of the water molecules were located in a difference Fourier map and refined as riding with the O—H bond lengths fixed to their as-found values and *U*_iso_(H) = 1.5 *U*_eq_(carrier O).

### Crystal data

**Table:**

[Cd(C_7_H_7_N_2_O_4_)_2_(H_2_O)_2_]·2H_2_O	*F*(000) = 1112
*M**_r_* = 550.76	*D*_x_ = 1.731 Mg m^−^^3^
Monoclinic, *C*2/*c*	Mo *K*α radiation, λ = 0.71073 Å
Hall symbol: -C 2yc	Cell parameters from 1702 reflections
*a* = 9.844 (2) Å	θ = 2.5–25.9°
*b* = 17.084 (3) Å	µ = 1.10 mm^−^^1^
*c* = 12.855 (3) Å	*T* = 293 K
β = 102.21 (3)°	Block, colourless
*V* = 2113.0 (8) Å^3^	0.30 × 0.25 × 0.18 mm
*Z* = 4	

### Data collection

**Table:**

Bruker SMART 1000 CCD area-detector diffractometer	1898 independent reflections
Radiation source: fine-focus sealed tube	1560 reflections with *I* > 2σ(*I*)
graphite	*R*_int_ = 0.042
φ and ω scans	θ_max_ = 25.2°, θ_min_ = 3.2°
Absorption correction: multi-scan (*SADABS*; Bruker, 2004)	*h* = −11→10
*T*_min_ = 0.733, *T*_max_ = 0.826	*k* = −20→20
8379 measured reflections	*l* = −15→15

### Refinement

**Table:**

Refinement on *F*^2^	Primary atom site location: structure-invariant direct methods
Least-squares matrix: full	Secondary atom site location: difference Fourier map
*R*[*F*^2^ > 2σ(*F*^2^)] = 0.028	Hydrogen site location: inferred from neighbouring sites
*wR*(*F*^2^) = 0.072	H-atom parameters constrained
*S* = 1.23	*w* = 1/[σ^2^(*F*_o_^2^) + (0.0164*P*)^2^ + 5.0177*P*] where *P* = (*F*_o_^2^ + 2*F*_c_^2^)/3
1898 reflections	(Δ/σ)_max_ < 0.001
142 parameters	Δρ_max_ = 0.77 e Å^−^^3^
6 restraints	Δρ_min_ = −0.78 e Å^−^^3^
0 constraints	

### Fractional atomic coordinates and isotropic or equivalent isotropic displacement parameters (Å^2^)

**Table:**

	*x*	*y*	*z*	*U*_iso_*/*U*_eq_	
Cd1	0.0000	0.20278 (3)	0.2500	0.03340 (14)	
O1	0.5880 (2)	0.14665 (16)	0.0777 (2)	0.0399 (7)	
O2	0.5052 (3)	0.26839 (16)	0.0774 (2)	0.0439 (7)	
O3	0.3063 (3)	0.33738 (16)	0.1245 (2)	0.0470 (7)	
H3	0.3668	0.3140	0.1034	0.071*	
O4	0.1229 (2)	0.30656 (16)	0.1896 (2)	0.0414 (6)	
N1	0.3597 (3)	0.08847 (17)	0.1520 (2)	0.0288 (7)	
H9	0.4044	0.0442	0.1391	0.035*	
N2	0.1837 (3)	0.14937 (18)	0.1946 (2)	0.0287 (7)	
C1	0.2678 (3)	0.2036 (2)	0.1605 (2)	0.0244 (7)	
C2	0.3782 (3)	0.1654 (2)	0.1332 (3)	0.0266 (8)	
C3	0.2420 (3)	0.0805 (2)	0.1895 (3)	0.0311 (8)	
C4	0.1894 (4)	0.0046 (3)	0.2202 (4)	0.0506 (11)	
H4A	0.2676	−0.0260	0.2578	0.061*	
H4B	0.1289	0.0145	0.2693	0.061*	
C5	0.1137 (8)	−0.0418 (4)	0.1323 (6)	0.127 (3)	
H5A	0.0380	−0.0115	0.0927	0.190*	
H5B	0.0780	−0.0880	0.1595	0.190*	
H5C	0.1750	−0.0565	0.0867	0.190*	
C6	0.5001 (3)	0.1949 (2)	0.0929 (3)	0.0316 (8)	
C7	0.2289 (3)	0.2871 (2)	0.1586 (3)	0.0320 (8)	
O1W	−0.1380 (3)	0.1871 (2)	0.0846 (2)	0.0691 (11)	
H2W	−0.2184	0.1706	0.0594	0.104*	
H1W	−0.1026	0.2090	0.0386	0.104*	
O2W	−0.0139 (3)	0.45120 (19)	0.1075 (3)	0.0695 (10)	
H4W	0.0458	0.4235	0.1478	0.104*	
H3W	−0.0307	0.4344	0.0448	0.104*	

### Atomic displacement parameters (Å^2^)

**Table:**

	*U*^11^	*U*^22^	*U*^33^	*U*^12^	*U*^13^	*U*^23^
Cd1	0.02313 (19)	0.0344 (3)	0.0474 (2)	0.000	0.01810 (16)	0.000
O1	0.0283 (13)	0.0410 (18)	0.0557 (16)	−0.0001 (12)	0.0208 (12)	−0.0051 (13)
O2	0.0461 (15)	0.0289 (18)	0.0667 (18)	−0.0072 (12)	0.0345 (14)	0.0007 (13)
O3	0.0492 (16)	0.0276 (16)	0.075 (2)	−0.0021 (13)	0.0379 (15)	0.0003 (14)
O4	0.0338 (13)	0.0300 (16)	0.0671 (17)	0.0057 (12)	0.0261 (13)	0.0010 (14)
N1	0.0266 (14)	0.0189 (17)	0.0439 (17)	0.0022 (12)	0.0143 (13)	−0.0014 (13)
N2	0.0244 (14)	0.0242 (18)	0.0404 (16)	−0.0010 (12)	0.0134 (13)	0.0001 (13)
C1	0.0238 (15)	0.0191 (18)	0.0320 (17)	−0.0039 (15)	0.0098 (14)	−0.0003 (15)
C2	0.0210 (15)	0.030 (2)	0.0307 (18)	−0.0007 (14)	0.0088 (14)	−0.0007 (15)
C3	0.0261 (17)	0.028 (2)	0.041 (2)	0.0000 (15)	0.0115 (16)	0.0016 (16)
C4	0.041 (2)	0.032 (3)	0.084 (3)	0.0013 (18)	0.026 (2)	0.013 (2)
C5	0.173 (7)	0.092 (6)	0.123 (6)	−0.090 (6)	0.049 (5)	−0.033 (5)
C6	0.0250 (17)	0.039 (3)	0.0337 (19)	−0.0047 (17)	0.0123 (15)	−0.0069 (18)
C7	0.0298 (17)	0.030 (2)	0.0383 (19)	−0.0029 (16)	0.0123 (16)	0.0000 (17)
O1W	0.0318 (14)	0.134 (4)	0.0430 (16)	−0.0229 (18)	0.0119 (13)	0.0104 (19)
O2W	0.085 (2)	0.049 (2)	0.068 (2)	0.0309 (18)	0.0014 (18)	−0.0144 (17)

### Geometric parameters (Å, °)

**Table:**

Cd1—N2^i^	2.270 (3)	N2—C1	1.375 (4)
Cd1—N2	2.270 (3)	C1—C2	1.375 (5)
Cd1—O1W	2.284 (3)	C1—C7	1.476 (5)
Cd1—O1W^i^	2.284 (3)	C2—C6	1.491 (5)
Cd1—O4	2.368 (3)	C3—C4	1.481 (5)
Cd1—O4^i^	2.368 (3)	C4—C5	1.450 (7)
O1—C6	1.239 (4)	C4—H4A	0.9700
O2—C6	1.275 (5)	C4—H4B	0.9700
O3—C7	1.284 (4)	C5—H5A	0.9600
O3—H3	0.8096	C5—H5B	0.9600
O4—C7	1.239 (4)	C5—H5C	0.9600
N1—C3	1.353 (4)	O1W—H2W	0.8388
N1—C2	1.356 (5)	O1W—H1W	0.8355
N1—H9	0.9080	O2W—H4W	0.8424
N2—C3	1.317 (5)	O2W—H3W	0.8381
			
N2^i^—Cd1—N2	132.60 (15)	N1—C2—C6	122.5 (3)
N2^i^—Cd1—O1W	83.59 (10)	C1—C2—C6	131.7 (3)
N2—Cd1—O1W	91.00 (11)	N2—C3—N1	110.1 (3)
N2^i^—Cd1—O1W^i^	91.00 (11)	N2—C3—C4	126.1 (3)
N2—Cd1—O1W^i^	83.59 (10)	N1—C3—C4	123.9 (3)
O1W—Cd1—O1W^i^	166.5 (2)	C5—C4—C3	114.9 (4)
N2^i^—Cd1—O4	154.13 (10)	C5—C4—H4A	108.5
N2—Cd1—O4	72.67 (10)	C3—C4—H4A	108.5
O1W—Cd1—O4	91.54 (10)	C5—C4—H4B	108.5
O1W^i^—Cd1—O4	98.55 (11)	C3—C4—H4B	108.5
N2^i^—Cd1—O4^i^	72.67 (10)	H4A—C4—H4B	107.5
N2—Cd1—O4^i^	154.13 (10)	C4—C5—H5A	109.5
O1W—Cd1—O4^i^	98.55 (11)	C4—C5—H5B	109.5
O1W^i^—Cd1—O4^i^	91.54 (10)	H5A—C5—H5B	109.5
O4—Cd1—O4^i^	83.03 (12)	C4—C5—H5C	109.5
C7—O3—H3	108.4	H5A—C5—H5C	109.5
C7—O4—Cd1	115.4 (2)	H5B—C5—H5C	109.5
C3—N1—C2	108.6 (3)	O1—C6—O2	125.3 (3)
C3—N1—H9	117.7	O1—C6—C2	118.1 (4)
C2—N1—H9	133.5	O2—C6—C2	116.6 (3)
C3—N2—C1	106.7 (3)	O4—C7—O3	122.1 (4)
C3—N2—Cd1	139.4 (2)	O4—C7—C1	119.1 (3)
C1—N2—Cd1	113.7 (2)	O3—C7—C1	118.8 (3)
N2—C1—C2	108.9 (3)	Cd1—O1W—H2W	136.5
N2—C1—C7	119.0 (3)	Cd1—O1W—H1W	110.6
C2—C1—C7	132.1 (3)	H2W—O1W—H1W	112.2
N1—C2—C1	105.8 (3)	H4W—O2W—H3W	111.6

Symmetry codes: (i) −*x*, *y*, −*z*+1/2.

### Hydrogen-bond geometry (Å, °)

**Table:**

*D*—H···*A*	*D*—H	H···*A*	*D*···*A*	*D*—H···*A*
O3—H3···O2	0.81	1.66	2.468 (4)	172.
O2W—H4W···O4	0.84	2.16	2.904 (4)	147.
O2W—H3W···O1^ii^	0.84	2.08	2.874 (4)	157.
O1W—H1W···O2^ii^	0.84	1.97	2.788 (4)	165.
O1W—H2W···O1^iii^	0.84	2.01	2.768 (3)	150.
N1—H9···O2W^iv^	0.91	1.86	2.771 (4)	177.

Symmetry codes: (ii) −*x*+1/2, −*y*+1/2, −*z*; (iii) *x*−1, *y*, *z*; (iv) *x*+1/2, *y*−1/2, *z*.
